# Mechanical and Time-Dependent Stability of K18-IBOA-Modified 3D-Printed Denture Base Resins

**DOI:** 10.3390/ma19122430

**Published:** 2026-06-06

**Authors:** Gregory Bennett, Mark Beatty, Bobby Simetich

**Affiliations:** Department of Adult Restorative Dentistry, College of Dentistry, University of Nebraska Medical Center, Lincoln, NE 68583, USA; mbeatty@unmc.edu (M.B.); bsimetic@unmc.edu (B.S.)

**Keywords:** 3D printing, denture base resin, antimicrobial, quaternary ammonium silane, K18-IBOA, flexural strength, mechanical properties, temporal stability

## Abstract

The incorporation of antimicrobial additives into 3D-printed denture base resins is a promising strategy for reducing denture stomatitis, but the mechanical consequences and temporal stability of such modifications remain poorly characterized. This in vitro pilot study evaluated the effects of incorporating 4% (*w*/*w*) K18—a quaternary ammonium methacryloxy silane dissolved in isobornyl acrylate (IBOA)—into three commercially available photopolymer resins printed across four platforms representing stereolithography, digital light processing, and liquid crystal display technologies. Flexural strength, flexural modulus, and flexure at break were measured at three time points: without K18, immediately after mixing, and one-week post-mixing. K18-IBOA incorporation produced significant reductions in all mechanical parameters across most resin–printer combinations. The magnitude and direction of changes were strongly dependent on printer–resin combination interactions, precluding generalization across systems. At one week, visible clumping consistent with phase separation was observed in all formulations, and one resin–printer combination exhibited catastrophic structural failure upon removal from the build platform. These findings suggest that 4% *w*/*w* K18-IBOA additions are unlikely to yield clinically acceptable denture bases without further formulation optimization, underscoring the importance of temporal stability assessment in the development of antimicrobial-modified photopolymer resins.

## 1. Introduction

### 1.1. Clinical Burden and Rationale for Antimicrobial Denture Materials

Complete tooth loss (edentulism) currently affects approximately 353 million people worldwide and represents a major global public health burden, disproportionately concentrated among older adults, women, and populations in low- and middle-income countries [1]. Edentulous patients depend on removable dentures as the primary prosthetic solution, and their long-term oral and systemic health outcomes are substantially influenced by the biological and mechanical performance of denture materials.

Denture surfaces provide an ideal substrate for microbial colonization. The accumulation of polymicrobial biofilms, dominated by *Candida albicans* and streptococcal species, are associated with denture stomatitis, a condition affecting an estimated 15–70% of denture wearers [2]. Beyond local oral disease, denture biofilm serves as a reservoir of respiratory pathogens, and overnight denture wear has been associated with an approximately 2.4-fold increased risk of pneumonia hospitalization or death in community-dwelling elderly adults [3]. Aspiration pneumonia represents one of the leading causes of infection-related mortality in elderly denture wearers [4].

The incorporation of antimicrobial agents directly into denture base materials has therefore emerged as a promising strategy to reduce biofilm formation at the material surface and improve long-term oral health outcomes in edentulous patients [5].

### 1.2. Antimicrobial Additives in Denture Base Resins: Effects and Limitations

Numerous studies have investigated the incorporation of bioactive agents into polymethyl methacrylate (PMMA)-based denture base resins, including silver nanoparticles [6,7,8], zinc-containing compounds [9], titanium dioxide nanoparticles [10], and polyethylenimine-based materials [11]. While many of these studies demonstrate antimicrobial activity in vitro, a consistent finding across the literature is that efficacy depends heavily on the dispersion and spatial distribution of the additive within the polymer matrix [12,13].

When particulate additives are incorporated as fillers, inadequate mixing or incompatible surface chemistry can promote agglomeration driven by van der Waals forces and high surface energy [14,15,16]. Agglomerated particles reduce effective antimicrobial surface area and create stress-concentrating defects within the polymer network, which may compromise both antimicrobial performance and mechanical integrity, particularly at higher filler loadings [13,15,17,18].

### 1.3. Quaternary Ammonium Silane Chemistry and the K18-IBOA Additive

Quaternary ammonium methacryloxy silicate (QAMS) compounds represent an alternative approach to antimicrobial functionalization of polymer systems. QAMS belongs to the class of organically modified silicates (ORMOSIL). Water-insoluble hybrid molecules containing multiple methacryloxy groups are attached to a siloxane backbone bearing a cationic quaternary ammonium nitrogen [19]. The quaternary ammonium moiety disrupts microbial cell membranes on contact, while the methacryloxy groups enable direct copolymerization into the polymer network upon light activation, covalently anchoring the antimicrobial function within the matrix rather than releasing it as a soluble agent. Supplied commercially as a liquid concentrate dissolved in methyl methacrylate (MMA), K18 can be blended with methacrylate-based monomers and co-polymerized directly into the resin [6,20]. Because the antimicrobial functionality is covalently integrated into the polymer matrix rather than physically dispersed as a discrete filler phase, QAMS-based additives offer durable, contact-mediated antimicrobial activity without reliance on ion leaching.

The K18 variant of QAMS has been incorporated into a range of dental materials with consistent antimicrobial efficacy against oral pathogens, though mechanical effects have varied by substrate and formulation. In orthodontic acrylic, QAMS incorporation yielded contact-killing activity against *S. mutans* and *C. albicans* with improved fracture toughness and no adverse effect on flexural strength or modulus [19]. In dental composites, K18-MMA combined with K18-functionalized filler produced significant antimicrobial activity against *S. mutans*, *S. sanguinis*, and *C. albicans*, with comparable elastic modulus to controls, though ultimate transverse strength was reduced in most K18-Filler-containing groups [21]. In denture liners, K18-modified formulations achieved hardness and modulus values comparable to or exceeding commercial controls, with significant antimicrobial efficacy, though elevated water sorption was observed at higher K18 concentrations [22,23]. Across these substrates, the mechanical impact of K18 incorporation appears to be material- and concentration-dependent, with no universal pattern of improvement or degradation.

Of particular relevance to the present study, Patel et al. incorporated K18 into a 3D-printable denture base resin both as a silane-functionalized filler and as a methyl methacrylate additive, reporting sustained antimicrobial activity with minimal changes in mechanical properties [24]. The K18-IBOA formulation evaluated in the present study differs from prior K18 investigations in that isobornyl acrylate (IBOA), rather than methyl methacrylate, serves as the vehicle monomer for K18 delivery.

### 1.4. 3D Printing for Denture Fabrication: Current Landscape and Limitations

Three-dimensional printing technologies used in dental prosthetics rely on vat photopolymerization, in which liquid photopolymer resins are selectively cured layer by layer through exposure to a light source. The three modalities for this study, stereolithography (SLA), digital light processing (DLP), and liquid crystal display (LCD), differ primarily in their light delivery mechanism: SLA employs a point-scanning laser, DLP projects each layer as a single image using a digital micromirror device, and LCD uses a pixel-masked backlight to define layer geometry [25,26]. Unlike conventionally heat-polymerized PMMA denture bases, 3D-printed denture base resins are typically formulated from urethane dimethacrylate (UDMA)-based photopolymer matrixes. Studies comparing 3D-printed and conventional denture bases have reported that 3D-printed materials generally achieve equivalent or lower flexural strength than milled CAD-CAM resins, with variability attributable to printing orientation, post-cure protocol, and resin–printer compatibility [27,28,29].

Despite these performance considerations, 3D printing offers compelling advantages for denture fabrication, including reduced chairside time, improved dimensional accuracy, and the ability to integrate functional additives directly into the liquid resin prior to printing [30]. Several investigators have exploited this property to develop antimicrobial 3D-printed denture base resins. Totu et al. [10] incorporated TiO2 nanoparticles into a stereolithographic PMMA resin and demonstrated antifungal activity against *Candida* species, though mechanical characterization was not a primary outcome. Taormina et al. [31] generated silver nanoparticles in situ within SLA resins and confirmed antimicrobial properties, while Kwon et al. [32] added zwitterionic compounds to 3D-printed PMMA, achieving durable biofilm resistance with minimal mechanical compromise; all materials in that study met ISO 20795-2 [33] requirements. Aati et al. [34] incorporated silver-loaded mesoporous silica nanoparticles into a 3D-printed resin, yielding improved hardness and fracture resistance, though flexural strength declined at concentrations ≥1 wt%. Jeon et al. [35] reported antifungal effects using phytoncide-filled microcapsules embedded in 3D-printed resin, at the cost of increased surface roughness. Across these studies, a consistent limitation is the failure to account for printer-to-printer variability. Each printing platform imposes distinct photopolymerization kinetics and energy dosing, meaning that additive effects observed with one system cannot be assumed to generalize to others [30].

### 1.5. Time-Dependence of Hybrid Resin Properties: Rationale for Temporal Evaluation

In clinical and laboratory settings, photopolymer denture base resins are commonly mixed with additives in advance and stored prior to printing, a workflow driven by batch preparation efficiency and equipment scheduling [30]. However, when a reactive silane compound such as K18-IBOA is introduced into a monomer system, the elapsed time between mixing and printing may be consequential. Reactive silane compounds bearing unsaturated methacryloxy groups are capable of undergoing partial pre-polymerization or silane condensation reactions in the presence of moisture or residual initiators, potentially altering the distribution and covalent availability of the additive before light exposure occurs [19]. Additionally, the density differential between the QAMS compound and the base monomer creates conditions favorable for sedimentation and phase separation during storage, a phenomenon documented for nanoparticulate and organosilicate additives in dental resin matrixes [12,14]. These physicochemical processes are time-dependent: the degree of redistribution or pre-reaction is expected to increase with storage duration, making the mechanical properties of the hybrid formulation a function not only of composition but of time elapsed since mixing. Although temporal stability has received limited attention in the dental literature, prior work demonstrated that time-dependent changes in printability and optical properties are detectable in modified 3D-printed denture base resins, underscoring the need for systematic temporal evaluation of hybrid formulations [36]. The one-week evaluation period in the present study was selected to represent a realistic delayed-printing scenario common in clinical and laboratory workflows.

### 1.6. Research Gap and Study Objectives

Efforts to develop antimicrobial denture base materials have employed both conventional processing methods [5,6,7,8,9,11,37] and emerging 3D printing technologies [10,31,32,35]. Although many of these studies report promising initial antimicrobial outcomes, the long-term mechanical stability of antimicrobial-modified resins remains incompletely characterized [5]. To our knowledge, no published study has systematically evaluated whether mechanical properties of K18-IBOA-modified 3D-printed resins change as a function of time elapsed between additive mixing and printing which is a scenario that reflects common clinical and laboratory workflows.

The objective of this in vitro pilot study was to evaluate the effect of incorporating 4% (*w*/*w*) K18 delivered in isobornyl acrylate into three commercially available 3D-printed denture base resins on flexural strength, flexural modulus, and flexure at break, as measured immediately after mixing and at one-week post-mixing. Four printing platforms representing the major photopolymerization technologies used in dental prosthetics were chosen. The null hypothesis was that K18-IBOA incorporation would not significantly affect flexural strength, flexural modulus, or flexure at break of 3D-printed denture base resins, and that mechanical properties would not differ between immediate and one-week post-mixing time points, regardless of printing platform (α = 0.05).

## 2. Materials and Methods

This in vitro pilot study evaluated the effects of incorporating a quaternary ammonium compound (K18-IBOA) on the mechanical properties of several 3D-printed denture base polymers over time. Specifically, the study assessed flexural strength, flexural modulus, and flexure at break of hybrid resin formulations printed immediately after mixing and at one-week post-mixing, as compared to unmodified resin.

### 2.1. Preparation of K18-Modified Denture Base Polymers

The K18-IBOA solution (FiteBac, Woodstock, GA, USA) was incorporated at 20% *w*/*w* of the final mixture. Given that K18 constitutes 20% of the K18-IBOA solution by weight, this yielded a final K18 concentration of 4% *w*/*w* in the printed resin, satisfying the manufacturer’s recommended threshold of approximately 5% K18 for antimicrobial efficacy and mechanical benefit in UV-cure materials [20]. This concentration is also consistent with that employed by Patel et al. [24], the most directly comparable prior study of K18 incorporation into a 3D-printable denture base resin. While prior K18 investigations have employed methyl methacrylate as the vehicle monomer, the present study used isobornyl acrylate, which is more chemically compatible with UDMA-based photopolymer systems. The 4% *w*/*w* concentration therefore represented the most clinically and experimentally justified starting point for this pilot investigation. The resin mixtures were prepared using three commercially available denture base resins: Denture Base Resin OP (Formlabs Inc., Somerville, MA, USA), DENTCA Denture Base II™ (DENTCA Inc., Los Angeles, CA, USA), and Apex Denture Base™ (SprintRay Inc., Los Angeles, CA, USA). The K18-IBOA was incorporated into each resin using a rolling mixer (LABFIDSH RM-6Pro, Huzhou City, China) at 80 RPM for 60 min. For each resin, a separate unmodified control group was prepared by printing the commercial resin without any K18-IBOA addition, using identical printing and post-processing protocols.

### 2.2. 3D Printing of Modified and Unmodified Resins

The K18-IBOA modified resins were printed using four different 3D printers, representative of the most common printing technologies in dentistry: one stereolithography (SLA) printer F3B (Formlabs Inc., Somerville, MA, USA), two digital light processing (DLP) printers S95 and 55S (SprintRay Inc.), and one liquid crystal display (LCD) printer S4K (Sonic Mini 4K, Phrozen Tech Co., Ltd., Hsinchu, Taiwan). All printers operated at a wavelength of 405 nm. The Form 3B uses a point-scanning laser rated at 250 mW; the Phrozen S4K uses an LED UV array with a peak power of 40 W. Light source power specifications for the SprintRay S95 and 55S are not disclosed by the manufacturer. For the Phrozen S4K, print parameters were set manually and included a layer exposure time of 5.2 s, lift distance of 5 mm, lift speed of 80 mm/min, bottom exposure time of 30 s, and retract speed of 150 mm/min. Print parameters for the Formlabs Form 3B and SprintRay S95 and 55S are managed by proprietary firmware and are not user-accessible or manufacturer-disclosed. Due to manufacturer resin–printer compatibility requirements, each printer was used exclusively with its designated resin: Denture Base OP (manufactured by DENTCA for Formlabs) with the F3B, DENTCA Denture Base II with the S95 and S4K, and Apex Denture Base with the 55S. As a result, printer and resin formulation were necessarily confounded, and observed differences between printers should be interpreted with this limitation in mind. Table 1 summarizes the printers, selected resins, and the resin manufacturers. Table 2 summarizes the resin and K18-IBOA formulations.

Rectangular bars were designed according to ISO 20795-1:2013 standards [33], with final dimensions of 64 mm length × 10 mm height × 3.3 mm thickness. The design was created in Meshmixer version 3.5.474 (Autodesk Inc., San Rafael, CA, USA) and exported as an STL file. Samples were printed at a 90° orientation on the build platform, without supports, using a 0.05 mm layer height. This was based on previous research, where optimal flexural properties were obtained for this printing configuration [38]. For each printer, specimens were printed from three mixture conditions: unmodified resin (no K18-IBOA), K18-IBOA-modified resin printed immediately after mixing, and K18-IBOA-modified resin printed at one-week post-mixing without any additional roller mixing. Ten specimens were produced per group, totaling 120 samples.

### 2.3. Post-Processing of Printed Samples

Post-processing protocols followed manufacturer recommendations [39]. Samples printed with Denture Base OP and DENTCA Denture Base II were washed in 91% isopropyl alcohol (IPA) for 30 min using a Form Wash unit (Formlabs Inc., Somerville, MA, USA), air dried, and post-cured at 405 nm in a 60 °C glycerin bath for 60 min using a Form Cure unit (Formlabs Inc., Somerville, MA, USA).

Apex samples were washed in 91% IPA for 9 min using a Prowash S unit (SprintRay Inc., Los Angeles, CA, USA), then air dried and post-cured for 9 min and 40 s in a Nano Cure unit (SprintRay Inc., Los Angeles, CA, USA). All samples were stored at 37 degrees Celsius in distilled water and protected from light until mechanical testing commenced.

### 2.4. Mechanical Testing

Prior to testing, all specimens were removed from storage, thoroughly dried, and labeled. Flexural testing was performed using a universal testing machine (Instron 5500R, Instron Corp., Norwood, MA, USA) configured for three-point bending. Each sample was placed on two supports 50 mm apart and loaded at its midpoint in compression at 5 mm/min until fracture occurred.

Flexural modulus (*E_f_*) was calculated using the following equation:

$$E_{f}=\frac{L^{3}}{4bd^{3}}\times \frac{\Delta P}{\Delta \delta }$$

where:

- *E_f_* is the flexural modulus (GPa)
- *L* is the support span (50 mm)
- *b* is the specimen width (mm)
- *d* is the specimen thickness (mm)
- Δ*P*/Δ*δ* is the slope of the linear portion of the load–displacement curve (N/mm)

Flexural strength (*S*) was calculated using the following equation:

$$S=\frac{3PL}{2bd^{2}}$$

where:

- *S* is the flexural strength (MPa)
- *P* is the maximum load at fracture (N)
- *L* is the support span (50 mm)
- *b* is the specimen width (mm)
- *d* is the specimen thickness (mm)

Flexure at break was recorded as the crosshead displacement at fracture (mm) and is reported as a measure of flexural deformation capacity prior to failure.

### 2.5. Statistical Analysis

Statistical analysis was performed using SPSS software (Version 29, IBM Corp., Armonk, NY, USA). Descriptive statistics, including minimum, maximum, mean, and standard deviation, were calculated for each group. Prior to analysis, the assumptions of normality and homogeneity of variance were evaluated for each dependent variable. Shapiro–Wilk tests indicated departures from normality in the majority of printer and mixture subgroups across all three outcomes. Levene’s test indicated violation of the homogeneity of variance assumption for flexural strength (F(11, 103) = 3.221, *p* < 0.001), flexural modulus (F(11, 103) = 3.096, *p* = 0.001), and flexure at break (F(11, 103) = 8.792, *p* < 0.001). Despite these violations, two-way ANOVA was retained given its known robustness to departures from normality and homogeneity of variance when cell sizes are adequate and the design is balanced [40]. Results should nonetheless be interpreted with appropriate caution, particularly for flexure at break, where variance heterogeneity was most pronounced. Separate two-way ANOVA tests with interaction were conducted for each dependent variable (flexural strength, flexural modulus, and flexure at break), with mixture (three levels: Unmodified, Immediate Print, and 1-Week Post-Mixing) and printer (four levels: F3B, S95, 55S, and S4K) as fixed factors. The mixture factor thereby captured both the effect of K18-IBOA incorporation and the effect of time elapsed between mixing and printing. Prior to conducting two-way ANOVA, non-parametric Kruskal–Wallis tests were performed on a combined printer-mixture grouping variable for each outcome. Statistically significant differences were detected across groups for flexural strength, flexural modulus, and flexure at break (all *p* < 0.001), consistent with the two-way ANOVA findings. As two-way non-parametric tests with interaction terms are not readily available, and the non-parametric results were consistent with the parametric analyses, the two-way ANOVA framework was retained. A sample size of n = 10 per group was selected to exceed the minimum specimen requirement of n = 5 specified in ISO 20795-1:2013 for flexural testing, and is consistent with sample sizes employed in recent similar studies of 3D-printed denture base resin mechanical properties [28,41,42]. Observed partial η^2^ values from each two-way ANOVA were converted to Cohen’s f and entered alongside the corresponding numerator degrees of freedom, total sample size (N = 120), and α = 0.05.

## 3. Results

Two-way analysis of variance (ANOVA) revealed significant effects of mixture, printer, and their interaction for all mechanical outcomes (all *p* < 0.001). Given the large Mixture × Printer interaction effects, results were interpreted at the level of simple effects rather than marginal main effects. Accordingly, estimated marginal means with Bonferroni-adjusted pairwise comparisons are presented to characterize mixture-specific differences within each printer and printer-specific differences within each mixture.

### 3.1. Modulus

**Figure 1:**
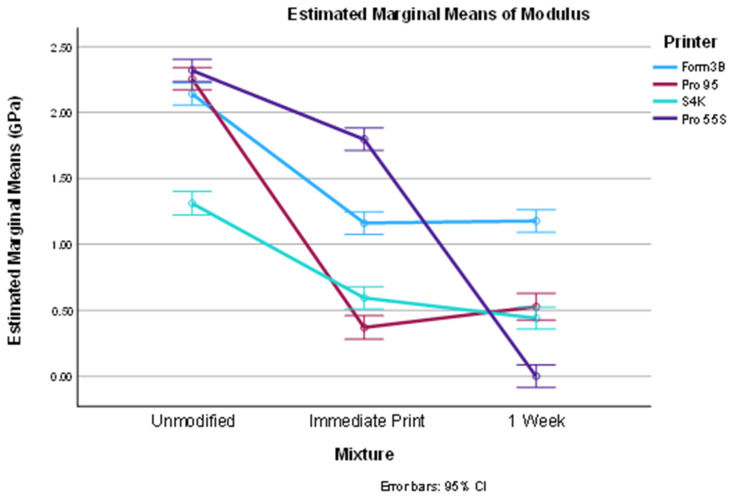
Estimated marginal means of flexural modulus for each printer at each time point.

Modulus was significantly influenced by mixture, printer, and their interaction (two-way ANOVA; Table 3). The large Mixture × Printer interaction effect indicates that modulus depended strongly on the specific printer/mixture combination rather than on either factor alone. Accordingly, estimated marginal means are presented in Table 4 and displayed in Figure 1 to illustrate simple effects. Within each printer, the unmodified mixture consistently exhibited higher modulus values than the Immediate Print and 1-Week conditions, although the magnitude of these differences varied by printer. Printer comparisons within each mixture further demonstrated substantial differences in modulus, with particularly pronounced reductions observed for the S4K resin–printer combination under all mixture conditions. Together, these results demonstrate that both mixture condition and resin–printer combination play a critical, interdependent role in determining material stiffness.

### 3.2. Flexural Strength

**Figure 2:**
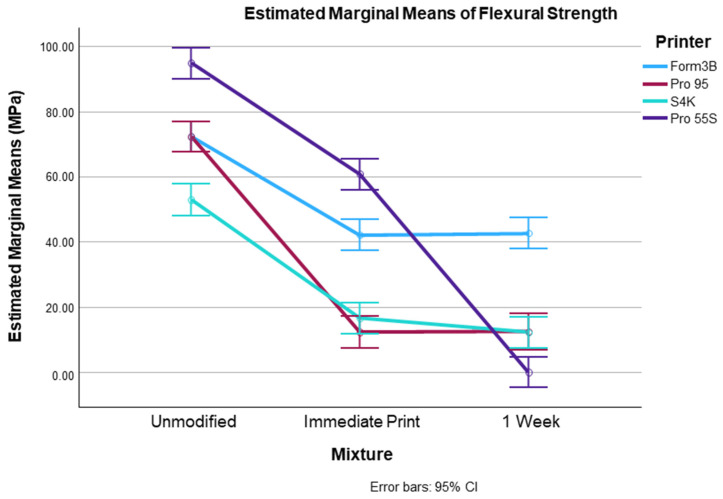
Estimated marginal means of flexural strength for each printer at each time point.

Flexural strength was also significantly affected by mixture, printer, and their interaction (Table 5). Interaction effects are summarized using estimated marginal means in Table 6 and displayed in Figure 2. Across all printers, the unmodified mixture yielded the highest flexural strength, while Immediate Print and 1-Week conditions generally showed reduced values. The extent of this reduction differed by printer. The 55S printer–resin combination demonstrated comparatively higher mean flexural strength across mixtures, with the Apex Denture Base maintaining 60.70 MPa at the Immediate Print time point compared to 12.16 MPa for the S95 and 16.46 MPa for the S4K, differences of approximately 48.5 and 44.2 MPa, respectively. By contrast, S95 and S4K exhibited pronounced decreases under modified conditions, with values remaining near these reduced levels at the one-week time point. Differences between resin–printer combinations within each mixture further underscore the dependence of flexural performance on both processing condition and resin–printer combination. With reference to the minimum flexural strength of 65 MPa specified by ISO 20795-1:2013 for denture base polymers, only the unmodified F3B, S95, and 55S resin–printer combinations met this threshold. The unmodified S4K resin–printer combination (52.94 MPa) fell below the ISO minimum even without K18-IBOA modification. No K18-IBOA-modified group met the ISO threshold at any time point.

### 3.3. Flexure at Break

**Figure 3:**
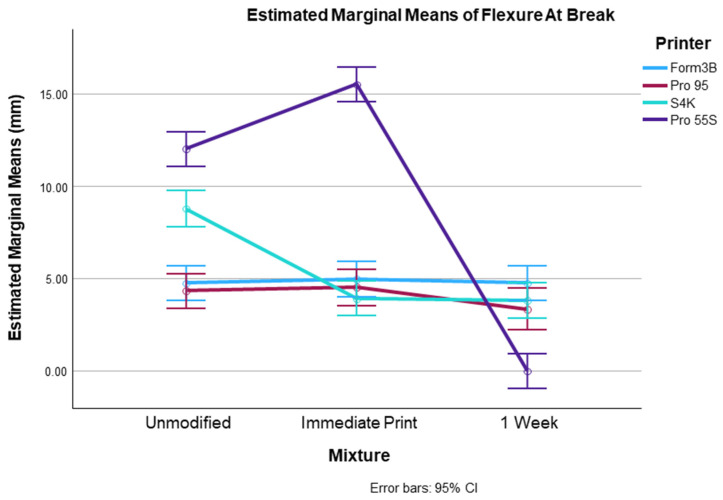
Estimated marginal means of flexure at break for each printer at each time point.

Flexure at break exhibited significant main effects and Mixture × Printer interaction (Table 7), indicating that deformation responses were dependent on printer/mixture combinations. Simple-effects results are presented in Table 8 and displayed in Figure 3 for F3B and S95 printers. Flexure at break did not differ significantly across mixture conditions, suggesting limited sensitivity to mixture modification. In contrast, S4K and 55S showed statistically significant mixture-dependent differences, with mean flexure at break values of 8.78 mm and 12.03 mm for the unmodified condition and 3.93 mm and 15.52 mm for the Immediate Print condition, respectively. This was followed by pronounced reductions at one week, with S4K decreasing to 3.82 mm and 55S specimens exhibiting catastrophic failure, precluding measurement. Printer comparisons within each mixture revealed that 55S resin–printer combination consistently exhibited the greatest flexure at break for unmodified and Immediate Print conditions. These findings indicate that flexure is sensitive to both resin–printer combination and mixture condition, with the Mixture × Printer interaction effect for extension at break (partial η^2^ = 0.805) exceeding that observed for flexural strength (partial η^2^ = 0.762), though remaining below that observed for modulus (partial η^2^ = 0.896).

## 4. Discussion

This project evaluated flexural properties of four resin–printer combinations before and after K18-IBOA additions. Properties were measured both immediately and one-week post-mixing. These four printers were selected to represent the range of photopolymerization platforms currently used in dental prosthetics fabrication, spanning dedicated dental-industry systems directly marketed to clinicians. These include Formlabs F3B, SprintRay S95 and 55S, and a low-cost consumer-grade LCD printer Phrozen Sonic Mini 4K, which has been increasingly adopted in dental settings due to its accessibility and affordability. Changes in material behavior following K18-IBOA addition over time was important because delayed printing commonly occurs, which causes a printing resin to sit for a period of time between printing jobs. The null hypothesis that K18-IBOA incorporation into 3D-printed denture base resins would not significantly affect flexural strength, flexural modulus, or flexure was partially rejected. The results demonstrated that all tested materials demonstrated significant reductions in the three flexural properties following the addition of K18-IBOA, with one exception. The exception was that the immediately-mixed Apex resin printed on the 55S printer exhibited improved flexure at break. The second null hypothesis, that mechanical properties would not differ between immediate and one-week post-mixing time points, regardless of printing platform, also was partially rejected. The 55S and S4K resin–printer combinations produced significantly lower mechanical properties over time, whereas the S95 and F3B resin–printer combinations underwent minimal mechanical change. Collectively these findings indicate that K18-IBOA incorporation altered mechanical behavior and, in several instances, reduced flexural strength below the minimum threshold of 65 MPa specified in ISO 20795-1:2013 for denture base polymers.

Two digital light processing (55S and S95), one stereolithography (Form 3B) and one liquid crystal display (S4K) printers were included in this study. The 55S and S95 DLP printers use digital projectors to cure each resin layer during denture build-up. Due to its speed, DLP technology is used for rapid prototyping. SLA printers (Form 3B) rely on a focused laser to polymerize resin continuously and precisely, which produce components with superior strength and fine detail. In contrast, LCD technology (S4K) is based on pixel arrays arranged on a LCD screen that cure resin in layers, which create polymers at lower cost and with higher surface roughness [43]. It should be noted, however, that these technology-based characterizations represent general descriptions of the printing modalities rather than conclusions drawn from the present data, as printer and resin formulation were necessarily confounded in this study.

In addition to dissimilarities in printing technologies, differences in resin compositions were expected to play a role in mechanical property differences. Table 2 shows manufacturer-reported compositions of the three polymers used in this study. For the 55S DLP printer, the Apex resin contains bismethacrylate, urethane dimethacrylate and monomeric methacrylate monomers, plus silicon dioxide filler. The resin is polymerized with a phosphine oxide initiator system. The monomers act to balance reactivity with viscosity. For the Apex resin, silicon dioxide filler provides additional reinforcement and improves strength, as neither of the other resins reports filler content on their SDS sheets. Phosphine oxide promotes rapid polymerization and has been reported to enhance the curing process, but also potentially reduces adhesion between layers [28,43]. The polymerized resin, with its high degree of crosslinking and silica filler reinforcement, is expected to exhibit high strength. This is confirmed in Table 6 and Figure 2, where the highest flexural strength of unmodified resins was achieved with the Apex resin printed by a 55S DLP printer. For the SLA printing resin (Denture Base OP), bisphenol A methacrylate and urethane dimethacrylate monomers are present and also commonly found in resin-based composites. When polymerized, these monomers produce materials with large molecular weight and high crosslink density. In this study, The SLA printer–resin combination produced flexural strengths that were intermediate to those measured for the other two printing resins. Table 2 does not show filler content being reported by the manufacturer of the SLA resin. If filler content is low or absent, this would account for lower strengths than those observed for the Apex resin. The resin printed by Pro 95 and LCD printers (DENTCA Denture Base II), contains methacrylate monomer, diurethane dimethacrylate and propylidynetrimethyl trimethacrylate. When polymerized, resins with lower molecular weight, crosslink density and higher shrinkage than the other two resins are expected. The mechanical properties are generally polymerized, particularly evident with the LCD printer, which produced polymers with low flexural modulus and strength.

When introduced into printing resins, K18 generally reduced the flexural properties of printed structures. K18 is a quaternary ammonium methacryloxy silane, and the silane core is capable of bonding with teeth and various materials. The N^+^ group contained in the quaternary ammonium group acts to disrupt cell membranes, thereby imparting antimicrobial activity. Three acrylate groups permit copolymerization with methacrylate monomers, and a C_18_ alkyl chain aids in penetrating bacterial cell walls. The long alkyl chain also can serve as a plasticizer, which lowers the glass transition temperature, modulus and strength of a polymer. Evidence of this is presented in Figure 1 and Figure 2, where all immediately-mixed resin–printer combinations demonstrated decreases in modulus and strength when compared to their unmixed counterparts. This effect was largely unchanged at one week for three of four resin–printer combinations.

**Figure 4:**
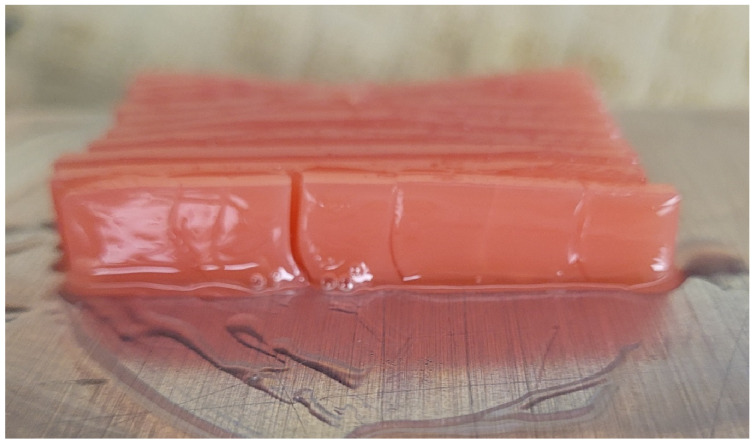
Shattered samples of Apex material printed on 55S at 1-week post-mixing.

Notably, the Apex Denture Base printed on the 55S printer, which produced the highest mechanical values at the immediate time point, exhibited catastrophic failure at the one-week time point. All ten specimens were successfully printed, yet each shattered under minimal pressure during removal from the build platform (Figure 4). This reversal, from best-performing to mechanically nonviable within a single week, is particularly striking. The catastrophic failure observed in the 55S/Apex combination at one week is not fully explained by phase separation alone, as visible clumping was observed across all resin formulations. The Apex resin is compositionally distinct from the resins used in the other printers in that it contains silica filler and a phosphine oxide photoinitiator, which are not confirmed in the other resins. One speculative possibility is that K18-IBOA may have interacted with the phosphine oxide initiator during storage, potentially depleting the initiator pool available for light-activated polymerization; however, this mechanism is not supported by direct chemical characterization in the present study and should be regarded as hypothesis-generating only. Direct investigation through degree of conversion measurements or FTIR analysis would be necessary to evaluate this possibility.

Prior work incorporating K18 into powder–liquid orthodontic acrylic formulations demonstrated enhanced fracture toughness, with no adverse effect on flexural strength or modulus [19,24]. However, the manufacturer’s claim [20] that approximately 5% K18 improves flexural, tensile, and anti-cracking properties in UV-cured resins was not supported by the flexural property results presented here. Instead, a significant decline in mechanical properties was observed immediately following K18-IBOA incorporation across all tested resin–printer combinations, with further degradation or catastrophic failure observed in several groups at the one-week post-mixing time point. A critical distinction is that the orthodontic acrylic system studied by Gong et al. [19] is a powder–liquid PMMA that is polymerized through chemical activation, whereas the resins evaluated in the present study are UDMA-based photopolymers, with fundamentally different monomer chemistries, network architectures, and crosslink densities that may respond differently to K18-IBOA incorporation. Whether K18-IBOA affects light transmission or degree of conversion in vat photopolymerization systems was not directly investigated in the present study and represents an important limitation. Bienek et al. [44] reported that incorporation of a quaternary ammonium silane with a shorter alkyl chain significantly reduced degree of vinyl conversion in a UDMA-based resin, whereas a longer-chain analog did not produce a statistically significant effect. By analogy, the influence of K18’s C18 alkyl chain on photopolymerization efficiency in the present systems is speculative, and degree of conversion measurements would be necessary before drawing mechanistic conclusions regarding network integrity.

Patel et al. [24] incorporated K18 into a 3D-printable denture base resin at 30% *w*/*w* in methyl methacrylate, diluted across a range of final concentrations. At a final K18 concentration of 5% *w*/*w*, no significant reduction in flexural strength was observed. The present study achieved a final K18 concentration of 4% *w*/*w*, yet significant mechanical degradation was observed across all resin–printer combinations. A direct comparison between these findings is complicated by the difference in vehicle monomer. Patel employed MMA, a small linear monomer, whereas the present study used IBOA, a bulky bicyclic monomer. Whether the mechanical degradation observed here is attributable to the K18 itself, to the IBOA vehicle, or to their combined interaction with the UDMA-based photopolymer matrix cannot be determined from the present data. Results reported by Kim et al. [45], also showed flexural strength reductions following combination of a 3D-printed UDMA denture base resin with a K18-containing monomer, suggesting that liquid-phase K18 delivery may be broadly capable of disrupting photopolymer network integrity. Improved understanding of this phenomenon requires additional research where the vehicle monomer and K18 concentration are independently varied within otherwise identical formulations.

Certain specimens in the present study, particularly the Apex resin printed on the 55S and the DENTCA resin printed on the S95, exhibited greater flexure at break concurrent with reduced flexural modulus. Such behavior may reflect a plasticizing effect of K18-IBOA within the polymer network. The plasticizing effect of K18-IBOA, evidenced by reduced modulus concurrent with significantly increased flexure at break, was observed only in the 55S/Apex combination at the immediate time point. The F3B and S95 combinations showed reduced modulus without a corresponding increase in flexure at break, suggesting general network disruption may have occurred, rather than classic plasticization. The S4K–resin combination showed immediate significant reductions across all three flexural properties, in addition to low values observed in the unmodified formulation. One possible contributing factor, though not directly confirmed in the present study, is the hydrophobic C18 alkyl chain of the K18 molecule. Long-chain alkyl groups are known to act as internal plasticizers in methacrylate-based polymer systems, and increased plasticization is generally accompanied by reductions in strength and modulus. Whether this mechanism accounts for the observed behavior would require direct characterization such as differential scanning calorimetry or dynamic mechanical analysis to confirm.

The resin mixing method may play a role in these outcomes. A rolling mixer was employed in this study to promote homogeneity while minimizing air incorporation. Prior studies provide limited descriptions of mixing protocols [23,24,32], using terms such as “shaken” [46,47], “stirred” [34,48,49], “mixed” [32,48], or “ultrasonicated” [10,34,49,50], with durations ranging from unreported [23,24,32,46,50], to 60 min [10,47], or longer [34,49]. The inconsistent reporting of mixing methodology limits reproducibility and complicates meaningful comparison across studies. This reinforces the need to standardize incorporation protocols.

**Figure 5:**
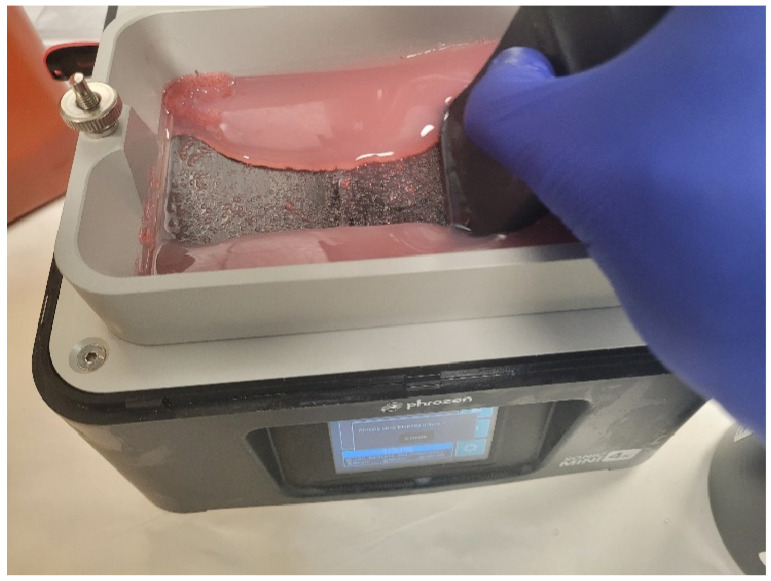
Visible clumping of resin at 1-week post-mixing.

A one-week printing trial was conducted to assess potential delayed effects of K18-IBOA incorporation on material stability. As outlined in the introduction, reactive silane compounds bearing unsaturated methacryloxy groups in K18-IBOA are capable of undergoing partial pre-polymerization or silane condensation reactions in the presence of moisture or residual initiators. Consequently, the density differential between the QAMS compound and the base monomer creates conditions favorable for sedimentation and phase separation during storage. Visible clumping was observed in all resin tanks at the one-week time point. While this observation is consistent with phase separation or partial pre-polymerization, these mechanisms were not directly characterized in the present study. Confirmation would require physicochemical analysis such as FTIR spectroscopy, rheological assessment, or microscopic examination, and the present observations should therefore be considered descriptive rather than mechanistically conclusive (Figure 5). Unlike Patel et al. [24], who reported clumping only at K18-MMA concentrations above 12.5%, clumping in the present study occurred at a final K18 concentration of 4%. This discrepancy may reflect the influence of the IBOA vehicle, which constitutes 16% of the final mixture and may independently contribute to phase instability during storage. The consequences of these changes were substantial and unpredictable, ranging from significant property reductions to catastrophic structural failure depending on the resin–printer combination. These findings indicate that K18-IBOA-modified denture base resins must be printed immediately following additive incorporation.

### Limitations

Study limitations include a small sample size (n = 10). Also, the inability to successfully remove printed specimens at the one-week time point for the 55S printer limited data collection for this group. Furthermore, the Formlabs Form 3B and SprintRay S95 and 55S operate as closed, proprietary systems in which both resin compatibility and print profiles are locked to manufacturer-validated materials via firmware controls, precluding cross-platform evaluation of individual resin formulations. Future studies should employ open-parameter platforms to enable true isolation of printer and material effects. While the operating wavelength of all printers is 405 nm, the light source power output for the SprintRay S95 and 55S is not publicly disclosed by the manufacturer, meaning the actual energy density delivered per layer during printing remains unknown for these systems. This represents an important limitation in directly comparing photopolymerization conditions across resin–printer combinations.

Additionally, only a single concentration of K18-IBOA (4% *w*/*w*) was evaluated. This concentration was selected based on the manufacturer’s recommended threshold for antimicrobial efficacy in UV-cure materials and is consistent with the concentration employed by Patel et al. [24] in the most directly comparable prior study of K18 incorporation into a 3D-printable denture base resin. The failures noted with this formulation may not apply to other concentrations or formulations. Concentration optimization, systematically titrating K18-IBOA upward or downward based on printability and mechanical performance, is identified as a priority direction for future work. Future research evaluating different antimicrobial additive concentrations and formulations, including systematic characterization of the time course of physicochemical degradation following mixing, is warranted. Additional storage intervals such as 24 h, 72 h, and one month would provide a more comprehensive understanding of degradation kinetics and practical workflow limitations; however, these are most meaningfully investigated once reformulation strategies that improve baseline stability have been identified. The present results provide the necessary foundation for prioritizing those investigations.

This investigation focused on flexural properties (flexural strength, flexural modulus, and flexure at break) which constitute the core mechanical requirements specified by ISO 20795-1:2013 for denture base polymers and are widely used as the first-line criterion for screening new or modified formulations. Flexural strength in particular functions as an initial go/no-go threshold: a material that fails to meet the 65 MPa minimum is unlikely to warrant the more resource-intensive characterization required for clinical qualification. Because the majority of K18-IBOA-modified resin–printer combinations did not meet this threshold, evaluation of additional clinically relevant parameters including fracture toughness, wear resistance, water sorption and solubility, fatigue behavior, and dimensional stability was considered premature for the present formulations and is identified as a priority for future work on any reformulated K18-IBOA system that first satisfies the ISO flexural requirement.

This study did not directly assess antimicrobial efficacy, and therefore no conclusions can be drawn regarding the balance between antimicrobial performance and mechanical behavior. Antimicrobial efficacy testing is an important next step in this line of investigation; however, given that the majority of K18-IBOA-modified resin–printer combinations failed to meet the ISO 20795-1:2013 minimum flexural strength threshold of 65 MPa, establishing mechanical adequacy represents a necessary prerequisite before antimicrobial performance can be meaningfully evaluated in a clinical context.

Taken together, the findings of this study reveal that the clinical translation of K18-IBOA-modified 3D-printed denture base resins faces at least three interdependent challenges: the consistent reduction in mechanical properties associated with additive incorporation, the strong dependence on printer–resin combination compatibility, and instability of formulations that prevents their use during delayed-printing workflows. No single resin–printer combination demonstrated both adequate mechanical performance and stability over time.

## 5. Conclusions

As a pilot in vitro investigation, this study provides an initial characterization of the mechanical and temporal stability of K18-IBOA-modified 3D-printed denture base resins across four resin–printer combinations. The findings should be interpreted within the scope and limitations of a pilot study, and generalization beyond the specific formulations and conditions tested requires further investigation.

Incorporation of 4% (*w*/*w*) K18 into 3D-printed denture base resins resulted in significant reductions in flexural strength, modulus, and extension at break for most of the resin–printer combinations evaluated. Although the Apex resin printed on the 55S printer demonstrated an increase in flexure at break immediately after mixing, this effect was not sustained. At one-week post-mixing, specimens exhibited catastrophic loss of structural integrity that prevented mechanical testing. These findings rejected the null hypotheses and demonstrated that K18-IBOA incorporation, under the conditions tested, consistently altered mechanical behavior in ways that are inconsistent with the minimum flexural strength requirements specified in ISO 20795-1:2013 for denture base polymers. These results pertain to the single 4% *w*/*w* concentration evaluated and should not be extrapolated to lower K18-IBOA concentrations, which were not tested. Whether these findings extend to other mechanical or clinical performance parameters requires further investigation.

Mechanical property changes were strongly dependent on the interaction between resin formulation and printing platform, showing that the effects of K18 additions cannot be generalized across photopolymer systems. The pronounced Mixture × Printer interactions observed in this study underscore the need to evaluate antimicrobial modifications within the specific resin–printer systems in which they will be clinically deployed.

A key finding of this investigation was the time-dependent instability of K18-IBOA-modified resins. Visible phase separation and delayed print failure observed at the one-week time point suggest limited time-dependent stability of the hybrid formulations, raising concerns of delayed-printing workflows that are common in clinical and laboratory environments. This short-term degradation may represent a barrier to the uniform adoption of antimicrobial-modified denture base resins.

## Figures and Tables

**Table 1:** Printers and materials used.

Printer	Technology	Resin	Resin Manufacturer
Form 3B (F3B) (Formlabs)	SLA	Denture Base OP	DENTCA (for Formlabs)
Pro 95 (S95) (SprintRay)	DLP	DENTCA Denture Base II	DENTCA
Pro 55S (55S) (SprintRay)	DLP	Apex Denture Base	SprintRay
Sonic 4K Mini (S4K) (Phrozen)	LCD	DENTCA Denture Base II	DENTCA

**Table 2:** Composition of denture base resins and K18-IBOA antimicrobial additive as reported in manufacturer Safety Data Sheets.

Resin	Components
SprintRay Apex Base (% *w*/*w*)	77,7,9(or 7,9,9)-trimethyl-4,13-dioxo-3,14-dioxa-5,12-diazahexadecane-1,16-diyl bismethacrylate (25–35%), Urethane Methacrylate (20–30%), Monomeric Methacrylate (15–25%), Asbestos free Silicon Dioxide (15–25%), diphenyl(2,4,6-trimethylbenzoyl)phosphine oxide (0.1–3%), Proprietary Ingredients (0.1–1%)
Formlabs Denture Base OP (% *w*/*w*)	Bisphenol A dimethacrylate (40–60%), Urethane dimethacrylate (30–50%), Methacrylate monomer (5–10%), Photoinitiator (<3%)
DENTCA Denture Base II (% *w*/*w*)	Methacrylate monomer (40–60%), Diurethane dimethacrylate (30–50%), Propylidynetrimethyl trimethacrylate (5–10%) Initator (≤3.0%), Inhibitor (≤1.0%), Pigment 1 (≤1.0%), Pigment 3 (≤1.0%), Pigment 5 (≤1.0%)
K18-IBOA (% *w*/*w*)	Isobornyl acrylate (80%), K18 (20%)

**Table 3:** Two-way ANOVA for modulus by mixture and printer.

Source	df	F	*p*	Partial η^2^
Mixture	2	1184.56	<0.001	0.958
Printer	3	165.92	<0.001	0.829
Mixture × Printer	6	148.27	<0.001	0.896
Error	103	—	—	—

Type III sums of squares were used. Partial η^2^ is reported as a measure of effect size. Model R^2^ = 0.974 (adjusted R^2^ = 0.971).

**Table 4:** Interaction effects of mixture and printer on modulus (GPa).

Printer	Unmodified	Immediate Print	1 Week
F3B	2.142 ± 0.043 ^aB^	1.161 ± 0.043 ^bB^	1.177 ± 0.043 ^bB^
S95	2.257 ± 0.043 ^aB^	0.368 ± 0.045 ^bD^	0.526 ± 0.051 ^bC^
S4K	1.312 ± 0.045 ^aA^	0.593 ± 0.043 ^bC^	0.439 ± 0.043 ^cC^
55S	2.321 ± 0.043 ^aC^	1.798 ± 0.043 ^bA^	~0.00 ± 0.043 ^cA^

Values are estimated marginal means ± standard error. Lowercase letters indicate significant differences among mixtures within each row (printer). Uppercase letters indicate significant differences among printers within each column (mixture). Means sharing the same letter do not differ significantly (*p* ≥ 0.05, Bonferroni-adjusted).

**Table 5:** Two-way ANOVA for flexural strength by mixture and printer.

Source	df	F	*p*	Partial η^2^
Mixture	2	564.6	<0.001	0.916
Printer	3	87.43	<0.001	0.718
Mixture × Printer	6	55	<0.001	0.762
Error	103	-	-	-

Type III sums of squares were used. Partial η^2^ is reported as a measure of effect size. Model R^2^ = 0.944 (adjusted R^2^ = 0.938).

**Table 6:** Interaction effects of mixture and printer on flexural strength (MPa).

Printer	Unmodified	Immediate Print	1 Week
F3B	**72.20** ± 2.38 ^aC^	42.09 ± 2.38 ^bB^	42.60 ± 2.38 ^bB^
S95	**72.28** ± 2.38 ^aC^	12.16 ± 2.50 ^bC^	12.39 ± 2.84 ^bC^
S4K	52.94 ± 2.50 ^aB^	16.46 ± 2.38 ^bC^	12.08 ± 2.38 ^bC^
55S	**94.77** ± 2.38 ^aA^	60.70 ± 2.38 ^bA^	~0.00 ± 2.38 ^cA^

Values are estimated marginal means ± standard error. Lowercase letters indicate significant differences among mixtures within each row (printer). Uppercase letters indicate significant differences among printers within each column (mixture). Means sharing the same letter do not differ significantly (*p* < 0.05, Bonferroni-adjusted). Values in bold exceed the minimum flexural strength threshold of 65 MPa specified by ISO 20795-1:2013 for denture base polymers.

**Table 7:** Two-way ANOVA for extension at break by mixture and printer.

Source	df	F	*p*	Partial η^2^
Mixture	2	104.3	<0.001	0.669
Printer	3	65.62	<0.001	0.657
Mixture × Printer	6	70.74	<0.001	0.805
Error	103	-	-	-

Type III sums of squares were used. Partial η^2^ is reported as a measure of effect size. Model R^2^ = 0.892 (adjusted R^2^ = 0.880).

**Table 8:** Interaction effects of mixture and printer on extension at break (mm).

Printer	Unmodified	Immediate Print	1 Week
F3B	4.76 ± 0.48 ^aC^	4.97 ± 0.48 ^aB^	4.76 ± 0.48 ^aB^
S95	4.34 ± 0.48 ^aC^	4.52 ± 0.50 ^aB^	3.34 ± 0.57 ^aB^
S4K	8.78 ± 0.50 ^aB^	3.93 ± 0.48 ^bB^	3.82 ± 0.48 ^bB^
55S	12.03 ± 0.48 ^aA^	15.52 ± 0.48 ^bA^	~0.00 ± 0.48 ^cA^

Values are estimated marginal means ± standard error. Lowercase letters indicate significant differences among mixtures within each row (printer). Uppercase letters indicate significant differences among printers within each column (mixture). Means sharing the same letter do not differ significantly (*p* < 0.05, Bonferroni-adjusted).

## Data Availability

The original contributions presented in this study are included in the article. Further inquiries can be directed to the corresponding author.

## Abbreviations

The following abbreviations are used in this manuscript:

K18-IBOA	K18 in isobornyl acrylate
SLA	Stereolithography
DLP	Digital light processing
LCD	Liquid crystal display
QAMS	Quaternary ammonium methacryloxy silane
